# A Dual Platform Approach to Transcript Discovery for the Planarian Schmidtea Mediterranea to Establish RNAseq for Stem Cell and Regeneration Biology

**DOI:** 10.1371/journal.pone.0015617

**Published:** 2010-12-14

**Authors:** Martin J. Blythe, Damian Kao, Sunir Malla, Joanna Rowsell, Ray Wilson, Deborah Evans, Jamie Jowett, Amy Hall, Virginie Lemay, Sabrina Lam, A. Aziz Aboobaker

**Affiliations:** 1 Deep Seq, Faculty of Medicine and Health Sciences, Queen's Medical Centre, University of Nottingham, Nottingham, United Kingdom; 2 Evolutionary Developmental Biology Laboratory, Centre for Genetics and Genomics, Queen's Medical Centre, University of Nottingham, Nottingham, United Kingdom; Universitat Pompeu Fabra, Spain

## Abstract

The use of planarians as a model system is expanding and the mechanisms that control planarian regeneration are being elucidated. The planarian *Schmidtea mediterranea* in particular has become a species of choice. Currently the planarian research community has access to this whole genome sequencing project and over 70,000 expressed sequence tags. However, the establishment of massively parallel sequencing technologies has provided the opportunity to define genetic content, and in particular transcriptomes, in unprecedented detail. Here we apply this approach to the planarian model system. We have sequenced, mapped and assembled 581,365 long and 507,719,814 short reads from RNA of intact and mixed stages of the first 7 days of planarian regeneration. We used an iterative mapping approach to identify and define de novo splice sites with short reads and increase confidence in our transcript predictions. We more than double the number of transcripts currently defined by publicly available ESTs, resulting in a collection of 25,053 transcripts described by combining platforms. We also demonstrate the utility of this collection for an RNAseq approach to identify potential transcripts that are enriched in neoblast stem cells and their progeny by comparing transcriptome wide expression levels between irradiated and intact planarians. Our experiments have defined an extensive planarian transcriptome that can be used as a template for RNAseq and can also help to annotate the *S. mediterranea* genome. We anticipate that suites of other 'omic approaches will also be facilitated by building on this comprehensive data set including RNAseq across many planarian regenerative stages, scenarios, tissues and phenotypes generated by RNAi.

## Introduction

Understanding how we might repair and regenerate lost or damaged organs is a major goal of biomedical research. However, an integrated understanding of how stem cells can be used to replace and repair damaged or ageing tissue remains, for the most part, a distant goal [1]. The planarian model system provides a simple model in which to understand how this process is regulated in a natural context [2]. These amazing animals are able to regenerate entire animals from small starting fragments after amputations and constantly replace their somatic tissues during homeostasis as part of their normal life history [3]. This ability can be traced to a population of collectively totipotent planarian adult stem cells (pASCs), classically called neoblasts [2].

During normal tissue turnover new stem cell division progeny replace older differentiated cells. Broad positional information is maintained and re-established during regeneration by conserved signaling pathways that perform analogous roles during the embryogenesis of other animals [4], [5], [6], [7], [8], [9], [10], [11], [12], [13], [14], [15]. Division progeny interpret these signals to ensure that they differentiate into the correct cells at the correct positions within the body. This homeostatic process is acutely responsive to nutrient conditions and planarians will grow and de-grow as they are fed and starved respectively [3]. On induction of regeneration by amputation, for example decapitation, the stem cell population undergoes a marked increase in proliferation throughout the wounded fragment(s) [16]. Stem cell progeny undergo precise differentiation to form the missing structures removed by amputation within seven days of the original injury, with the restoration of perfect scale and proportion taking around 14 days [2].

It is these two interdependent processes of ongoing homeostatic replacement and regenerative potential that make planarians a fascinating model system [17]. In recent years the inevitable application of modern molecular genetic techniques has made understanding the molecular mechanisms controlling regeneration a realistic goal. For this reason it seems timely to produce a high quality publicly accessible description of the transcribed genetic content of the model planarian *S. mediterranea*.

The development of massively parallel sequencing platforms has allowed a set of new biological questions to be asked [18]. In particular a number of recent studies have employed massively parallel sequencing to generate more complete descriptions of transcribed portions of genomes [19], [20], [21], [22], [23], [24]. The approaches that can be facilitated by these large datasets include expression screens [25], large functional screens [26], interactome [27] and phylogenetic studies with large datasets of homologous genes [28].

The genome of the planarian *S. mediterranea* has been sequenced using a whole genome shotgun approach. Assembly of reads representing in excess of 11.6 fold coverage has surprisingly resulted in 43,294 super-contigs, with assembly problems caused by a number of issues. The A/T rich nature of the genome (greater that 65%) and its apparent repetitiveness (more than 40%) clearly contribute to the current poor assembly metrics (see genome.wustl.edu for information on the *S. mediterranea* genome). In addition to the genome sequence there are also 74,388 publicly available EST sequences from cDNA libraries (although the clones themselves are not publicly available). Finally, some researchers have made an effort to create a useful annotated database for the community which contains 30,930 gene predictions using the MAKER pipeline [29], attempting to incorporate data from ESTs, BLAST homology and even from presumably ongoing RNAi screens and array based studies of gene expression [30], [31]. In order to facilitate a systems approach to understanding planarian generation we would like an as complete as possible description of the planarian transcriptome.

Here, we generate and describe a transcriptome from a mixed time course of regenerative stages of the asexual strain of the planarian *S. mediterranea* to use as a basis for genome wide expression analyses, such as RNAseq. We have integrated data from two next generation sequencing platforms in an attempt to increase gene discovery and transcript representation as well as avoid single platform bias. Our analysis highlights some of the current difficulties, and presents some simple solutions, with generating a dual platform transcriptome in relation to a fairly poorly assembled genome. We also show that the collection of transcripts we have defined is suitable for RNAseq approaches. We compare expression levels of genes in intact worms with those that have been irradiated to remove proliferating adult stem cells and their recent undifferentiated progeny. This defines the set of transcripts whose expression is potentially enriched in adult stem cells and their progeny.

## Results and Discussion

### Increased planarian gene discovery using 454 transcriptome sequencing

**Figure 1:**
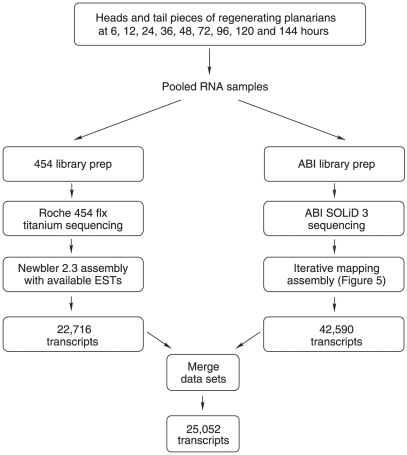
Overview of pipeline obtaining planarian transcripts. A schematic flow chart showing the work-flow for defining the planarian transcript set using a dual platform approach. 20 size-matched worms for each time point were cut into head, middle and tail pieces. Regenerating head, tail and middle fragments for RNA preparation were collected and frozen at 6, 12, 24, 36, 48, 72, 96, 120, 144 and 168 hours of regeneration. Total RNA was prepared from these time points, pooled and libraries made for both platforms.

**Figure 2:**
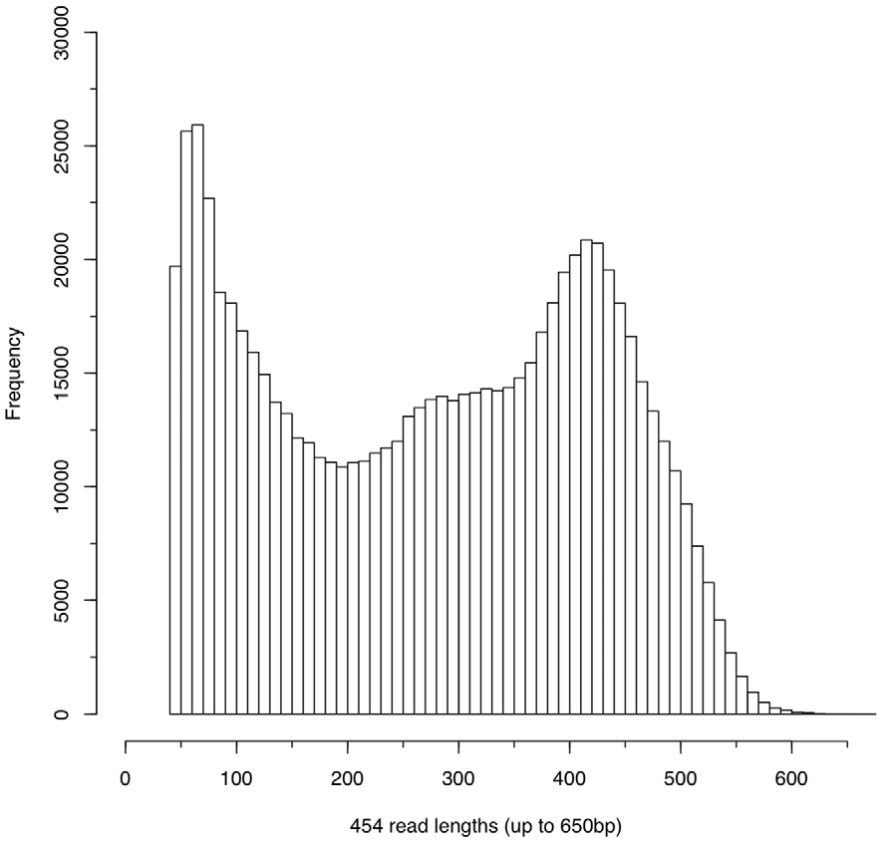
454 read length distribution. A plot showing the distribution of the raw planarian 454 reads generated from one pico-titre plate run with Titanium chemistry.

Currently planarian researchers are supported by the existence of a whole genome shotgun assembly and 74,388 ESTs for *Schmidtea mediterranea* [30], [32]. We wished to attempt to provide a more complete description of the transcriptome to enable a broader systems approach to understanding regeneration and adult stem cells in *S. mediterranea*. In order to do this we decided to sequence transcripts from whole and regenerating worms from different stages of the first 7 days of regeneration (see Materials and Methods and Figure 1). Combining equal amounts of input material from a mix of different stages we prepared a normalized 454 cDNA library. We sequenced from this library and generated 743,464 454 reads. These reads had a mean length of 278 bp, and an overall length distribution characteristic of 454 transcriptome sequencing with Titanium chemistry on this platform (Figure 2). Assembly detected 16,967 putative “isogroups” (genes) and a total of 21,030 putative “isotigs” (isoforms). We added 74,388 publicly available ESTs for *S. mediterranea* along with our 454 data. The combined data utilized 581,365 of the 454 reads in the assembly and resulted in 17,628 putative genes and 22,698 isoforms. We refer to this dataset as Sm454EST throughout this manuscript (Table 1 and Dataset S1).

**Table 1:** List of datasets.

Dataset	Number of transcripts
Sm454EST	22,716
SmABI	42,950
Sm454ESTABI	25,052
MAKER	30,930

**Figure 3:**
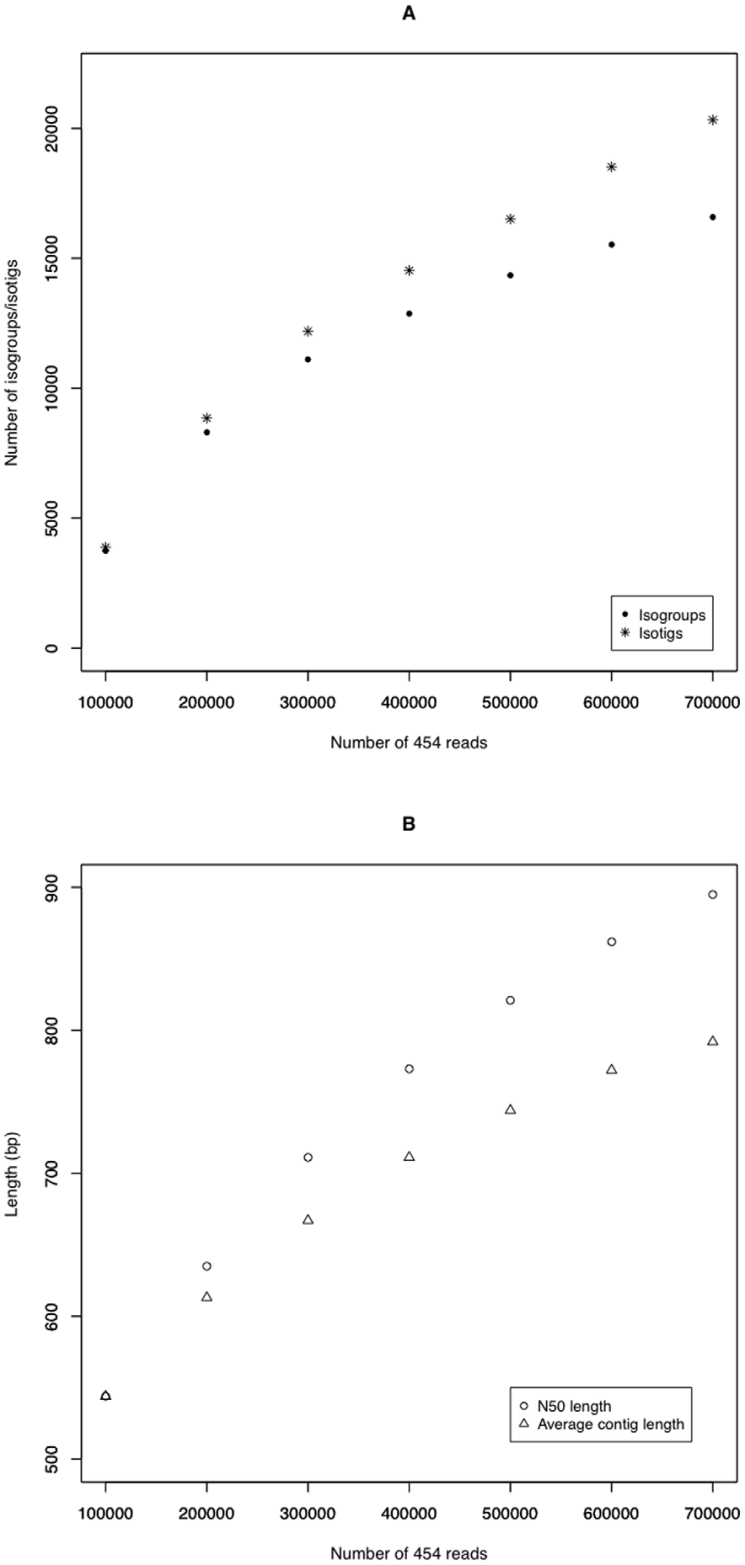
Increasing isogroup/isotig numbers and contig length with increasing read number. **A.** The number of Isotigs and Isogroups continue to increase with increasing number of reads added to the assembly, however Isogroup (gene) discovery appears to be a approaching a maximum whereas Isotig discovery (transcript isoforms) continues to increase. This suggests further sequencing would be beneficial to define more alternately spliced transcripts. **B.** The lengths of contigs also increase as more reads are added to the assembly. While many contigs are not any getting longer (average contig length) further sequencing is increasing the N50 perhaps by joining some transcripts together to form single contigs. Both analyses suggest that further sequencing of the mixed stage library may generate more transcripts and continue to increase the average length of contigs.

**Figure 4:**
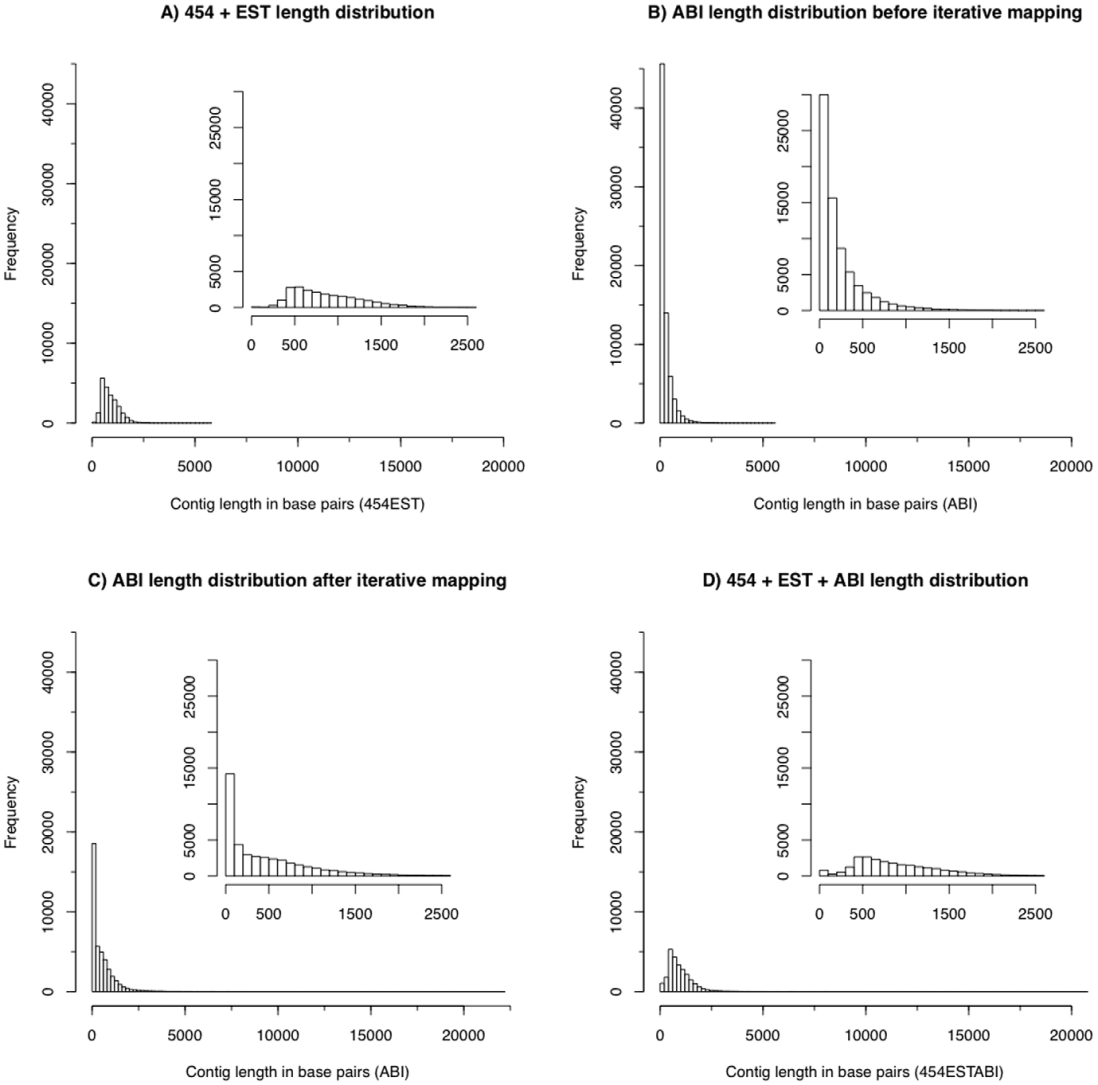
Contig length distributions. Contig length distribution for the following datasets (A) Sm454EST (B) SmABI before iterative mapping (C) SmABI after iterative mapping (D) Sm454ESTABI merged dataset. The subplots for each figure focus on contigs with length up to 2,500 bp. The short read approach using SOLiD clearly produces many short putative transcripts, many of these may be transcribed regions that do not encode proteins or which splice reads were not identified. Iterative mapping resulted in fewer short transcribed regions after filtering and longer contigs (B vs C) Combining the datasets led to an increase in contig lengths compared to either platform alone.

We analyzed how the number of predic ted transcripts (isogroups), predicted isoforms (isotigs), N50 length, and average contig length increased with increasing read number (Figure 3A and B) by assembling random selections of sequentially larger subsets of the total read set. These data sets suggest that even deeper sequencing with the Sm454 library would still yield significant novel transcript discovery and increases in average contig length (our 454 library is freely available on request). The length distribution of the contigs in this data set shows that most transcripts identified are over 400 bp in length (Fig 4A)

The current *S. mediterranea* genome database provides a set of 30,930 MAKER based [29], [30]; here we have called this data set SmMAKER (Dataset S2). We compared Sm454EST data set with SmMAKER by BLASTN [33]. We found that 18,797 SmMAKER annotations had a cognate Sm454EST hit, representing the same transcript (Table 2). Conversely we found that this represented 13,887 Sm454EST transcripts, suggesting some level of redundancy in the SmMAKER transcripts and that some annotations may be of duplicate sequences in the genome or separate gene predictions that are actually single genes. We found that 8,811 of 22,698 Sm454EST defined transcripts had no cognate MAKER annotations, immediately highlighting the benefits of transcriptome sequencing for gene discovery, and eventual annotation. We reasoned most must represent un-annotated transcripts or be from regions not in the current genome assembly. Mapping Sm454EST transcripts to the genome by BLASTN [33] or using GMAP [34] revealed that less than 2% failed to map across at least 80% of their length. This suggests that most of the 8,829 SM454EST transcripts without an SmMAKER match are un-annotated rather than missing from the current genome assembly. We found that 12,133 MAKER predictions had no cognate hit in the Sm454EST dataset suggesting that they are not transcribed genes or, just as likely, deeper sequencing is required to identify them (see below).

**Table 2:** Comparison of transcriptomes with existing datasets.

	454/EST	ABI	454/EST/ABI	MAKER
**454/EST**	x	16,374	22,682	13,887
**ABI**	28,618	x		30,223
**454/EST/ABI**	21,502		x	16,726
**MAKER**	18,797	20,745	22,189	x

### Massively parallel sequencing of short reads with iterative mapping further supplements gene discovery

Short read platforms offer the possibility of ultra-deep transcriptome coverage for both transcript discovery and the analysis of expression levels with unprecedented resolution [35], [36]. However, the use of short reads for the process of measuring genome wide expression levels, an approach called RNAseq, is somewhat dependent on the existence of high quality transcriptome annotations [23], [37], [38], [39]. Using short reads to define a transcriptome poses a number of problems. Firstly, a genome sequence is still required to achieve long contig lengths despite improvements in de novo short read assemblers [40]. Secondly, short reads will be prone to certain inaccuracies when used to define a transcriptome by mapping to a genome sequence. Examples include inaccurate mapping due to sequencing errors, mapping to regions that are not transcribed but have homology to transcribed regions (for example to pseudogenes) and by missing small exons and/or introns to which isolated sort reads fail to map.

**Figure 5:**
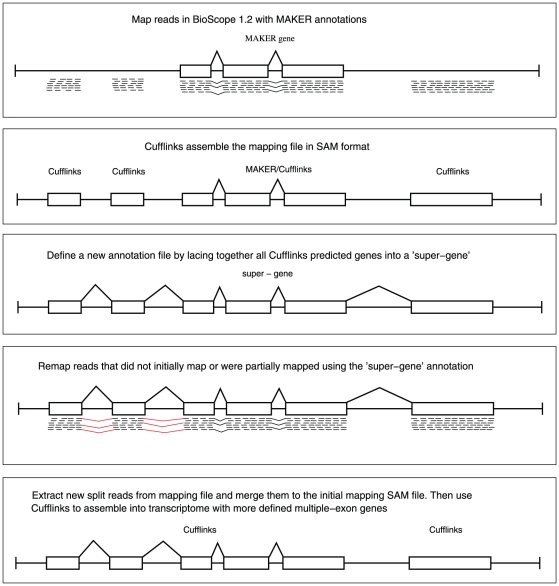
Defining transcript variants in more detail. A schematic flow chart showing the iterative mapping approach. New split reads found in the second round of mapping are in red.

In the case of *S. mediterannea* the genome assembly comprises 43,294 contigs with no chromosomal structure. To deal with this problem we used an iterative mapping approach to increase the definition of splice junctions between exons and define alternative transcript sequences (see Figure 5). We generated 903,642,430 50 bp reads using two flow cells on the SOLiD 3+ sequencing platform using a library prepared from the same mixed stage RNA pools (see materials and methods). We mapped reads using the existing published MAKER annotations from SmedGD as a starting point of our analysis [30]. We applied a simple filter to remove reads with low quality colour calls or low mapping scores from the resulting SAM file. We achieved high quality mapping of 507,719,814 reads, representing around 56% of the raw sequenced reads. Using the total length of the available MAKER annotations as an estimate this conservatively represents 1,060 fold coverage of the planarian transcriptome. We then used the Cufflinks program [41] to interpret the high quality mapped reads to produce a new annotated GTF (see materials and methods). Cufflinks was used to remove regions that were represented by very low read coverage and to define split-reads that crossed splice sites predicted by the SmMAKER annotations. This initial transcriptome GTF file comprised 19,429 putative multiple exon transcripts defined entirely by those genes that were in the original annotation file (i.e. in the SmMAKER dataset), and over 153,038 putative single exon transcripts. Many of these single exons reads were very short, with over 30,000 being consisting of two read lengths or less (Figure 4B).

The occurrence of mapped split-reads enables the definition of gene structure and different isoforms. To define as many split-reads as possible we remapped selected reads against the initial exon annotation defined by Cufflinks, but with exons grouped to form hypothetical “super-genes” on each strand of individual genomic contigs (see Figure 5). Of the 43,294 total contigs we were able to detect expression on only 15,897, representing 20,611 super-genes as not all contigs showed expression on both strands. A drawback of this approach is that low quality reads may erroneously map to splice junctions that are not real. Therefore we only remapped high quality reads that initially mapped giving a partial alignment string (cigar) possibly indicative of one half of a split-read. We found that this method defined many new splice junctions.

We then reassembled the new split-reads mappings in combination with the original mappings using Cufflinks. The number of multiple exon transcripts in the new assembly increased from 19,429 to 26,791 and the average length increased from 387 bp to 490 bp, reflected in the distribution of contig lengths (Figure 4C). This was a result of an increased number of multiple exon genes, additional isoform discovery, and of extension of transcripts defined in the first round of mapping. A total of 15,799 single exon transcripts were also retained to look for possible overlaps with the Sm454EST dataset in a merged assembly. We call this dataset SmABI (Dataset S3).

These data suggest that ultra-deep sequencing using short read technology clearly defines more transcribed regions than deep sequencing with 454 technology and may also define many more putative alternate splicing events. It is also likely that we failed to detect many splice events between contigs due to the fragmented nature of the planarian genome. We may have also detected many pseudogenes and duplicate regions unresolved in the current genome assembly, as short reads are able to map to accurately to these regions. Additionally, we might have detected non-coding transcripts of multiple origins and functions. We compared the SmABI dataset to the SmMAKER dataset and found that of the 42,950 putative SmABi transcripts 30,223 matched to the SmMAKER dataset, thus almost 30% of transcribed regions detected by this approach were not represented in previous annotations. When we looked for representation of the 30,920 SmMAKER annotations in the SmABi dataset we were able find similarity by BLASTN for 20,275. This is approximately 1300 more than were found in the Sm454EST dataset and maybe be the result of the deeper coverage achieved by SOLiD sequencing compared to 454 sequencing.

### An overview of planarian transcriptome structure from a combined platform approach

We wished to optimize our data by combining the long read and short read data sets. We extracted the cDNA sequences of the transcripts in SmABI and merged them with the Sm454EST data sets (see materials and methods). To combine the datasets we first assembled the Sm454EST data generated by GSAssembler with CAP3 [42] to collapse very similar isoforms into single transcripts. This resulted in 21,169 contigs that represented transcripts and isoforms with differences in internal exon content (isoforms with alternate terminal exons will have assembled). We then identified all SmABI contigs that matched with this data set by screening for significant hits with BLASTN (see materials and methods). This identified 27,830 SmABI contigs that had high BLASTN homology with the CAP3 assembled Sm454EST dataset. Finally, from those SmABI contigs that did not clearly overlap as indicated by BLASTN we excluded single exon genes and kept 6,494 multiple exon genes, likely to be real transcripts as they have spliced read evidence. Single exon SmABI genes without evidence from another source were excluded as they may represent duplications and pseudogenes to which short reads are still able to map. We then assembled the 21,169 contigs derived from the Sm454EST data with 6,494 unique multiple exon ABI contigs and 27,830 ABI contigs that had BLASTN matches to the Sm454EST dataset using CAP3. This resulted in 11,308 assembled contigs (combinations of two platforms) and 19,639 singletons (only represented by one platform). We removed 5,894 singletons representing single exon genes from the SmABI dataset, as we could not ascertain whether they represented real transcripts. This combined dataset Sm454ESTABI (Dataset S4) resulted in 25,053 transcripts that were defined by long read 454EST sequencing, a combination of platforms, or spliced transcripts identified by ABi SOLiD sequencing. The distribution of contig lengths of this dataset is longer than that for either dataset alone (Figure 4D).

We compared the Sm454ESTABI dataset to the SmMAKER annotation set (Table 2). We found that 22,189 MAKER annotations were now represented by 16,726 Sm454ESTABI transcripts. We interpret this as highlighting redundancy in the MAKER annotations. The combined dataset contained many more of the SmMAKER trascripts than the Sm454EST or SmABi dataset alone (Table 2), suggesting that gene discovery is increased by combining and assembling data from both platforms. The remaining approximately 8,000 SmMAKER annotations may not represent real genes or may represent transcribed regions that are expressed in life history stages we have not sampled in this work. It is also possible that some represent differences between the sexual and asexual transcriptome. We also found that 8,316 transcribed regions defined in Sm454ESTABI were absent from the SmMAKER annotations, indicating that we are likely to have identified a large number of novel planarian transcripts.

**Figure 6:**
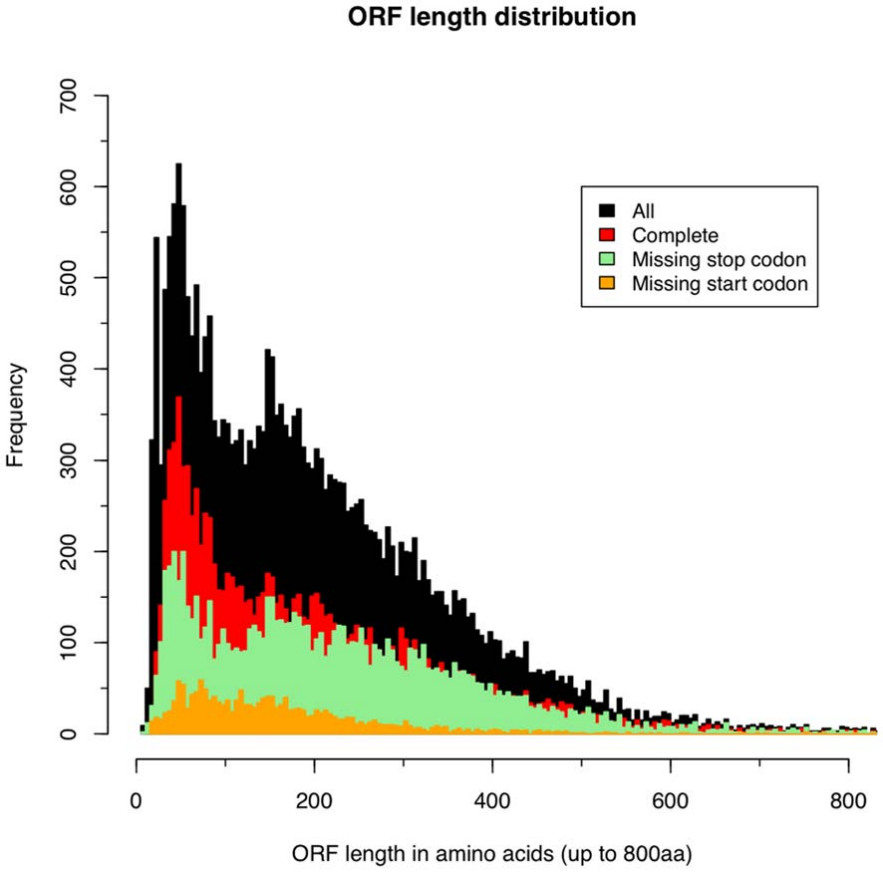
Open reading frame length distribution. Length distribution of the longest putative protein that could be coded for by each transcript for the Sm454ESTABI dataset (black). 11,599 open reading frames had both a putative start and stop codon (red), 9,076 missed the start (green) codon and 1,757 missed the stop codon (orange). The remaining 2620 transcripts either had neither a start or stop.

We performed a simple open reading frame analysis (ORF) on all of the Sm454ESTABI transcripts to identify the longest putative protein that could be coded for by each transcript file (Dataset S5). We found 11,599 putatively full-length ORFs with a possible start and stop codon, 9,076 with the start codon missing, and 1,757 with the stop codon missing. The remaining transcripts either did not have either a start or stop codon or no clear ORF (Figure 6). We assume that the large number of short ORFs with both starts and stop codons around the 50 amino acid size are probably not real proteins but represent non-coding regions of transcripts.

### Gene ontology, homology and novelty in the planarian transcriptome

In order to further validate and analyze the protein coding content of our Sm454ESTABI dataset we performed three independent analyses: 1. a cross-species BLASTX [33] analysis against the proteomes of 18 species. 2. transcript sequences were scanned for homology to Pfam protein families using Pfam-A hidden markov models v.24 [43]. 3. Gene Ontology (GO) database [44] classification analysis using the Blast2go pipeline v.2.3.5 [45] against a dataset consisting of the NCBI non-redundant invertebrate protein sequences, supplemented with sequences from the cross-species BLASTX analysis not included in the NCBI dataset. For all three analyses a lower significance threshold E-value of 1e-5 was applied for BLASTX.

**Figure 7:**
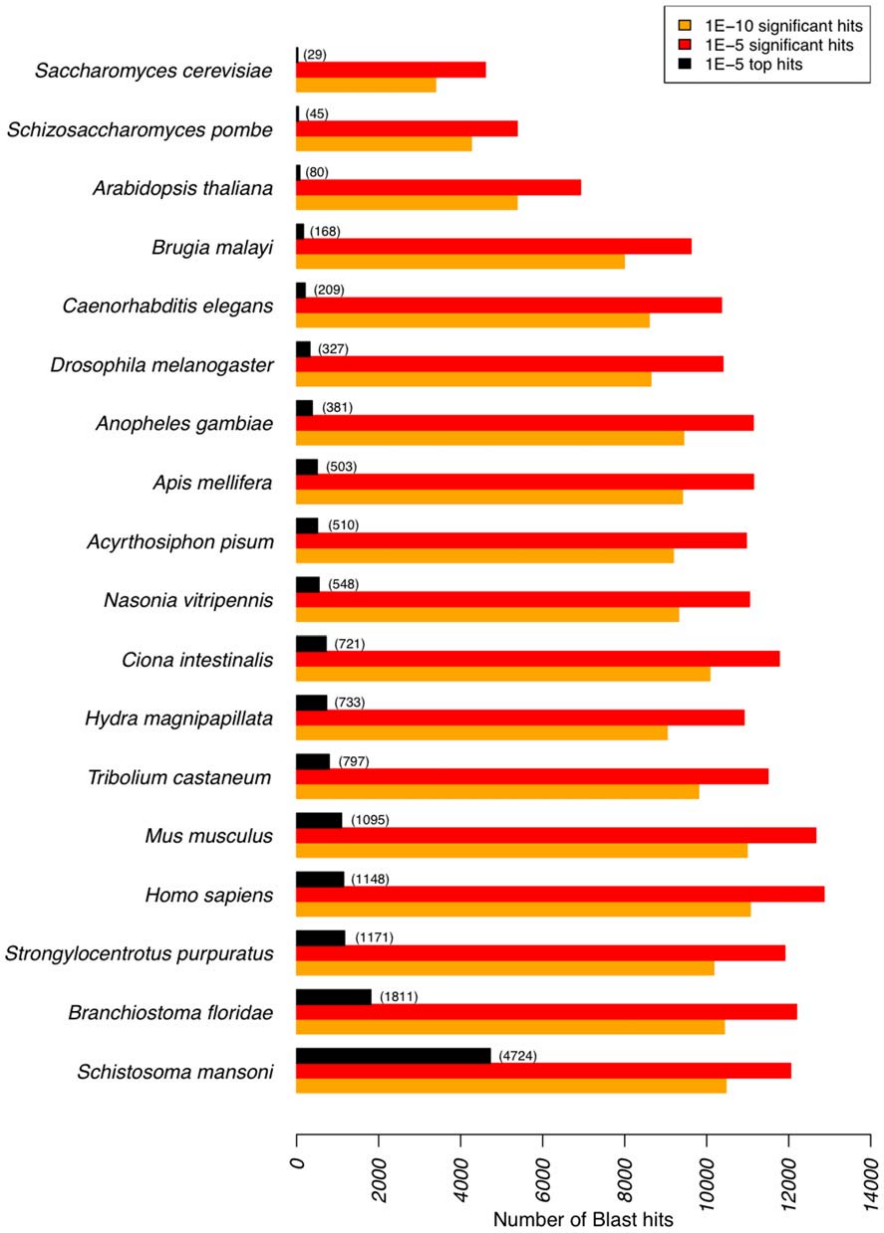
Cross-species BLASTX results. The BLASTX cross-species results for the Sm454ESTABI dataset. All species with a significant hit (probability < 1e-5 red bar, 1e-10 orange bar) are shown. Also shown are the number of times the top hit was to a particular species (black bar and number in brackets).

For the cross-species BLASTX analysis a total of 18,742 significant blast hits were calculated for 15,000 of the 25,053 transcripts (Figure 7, Dataset S6), while the remaining 10,053 showed no significant homology. Of the matched transcripts 915 showed homology to only a single species; more than half of these (479) to S. *mansoni*. Not surprisingly the most top hits were also to the *S. mansoni* genome. The number of top hits broadly followed the accepted phylogenetic distance of planarians from the compared species.

**Figure 8:**
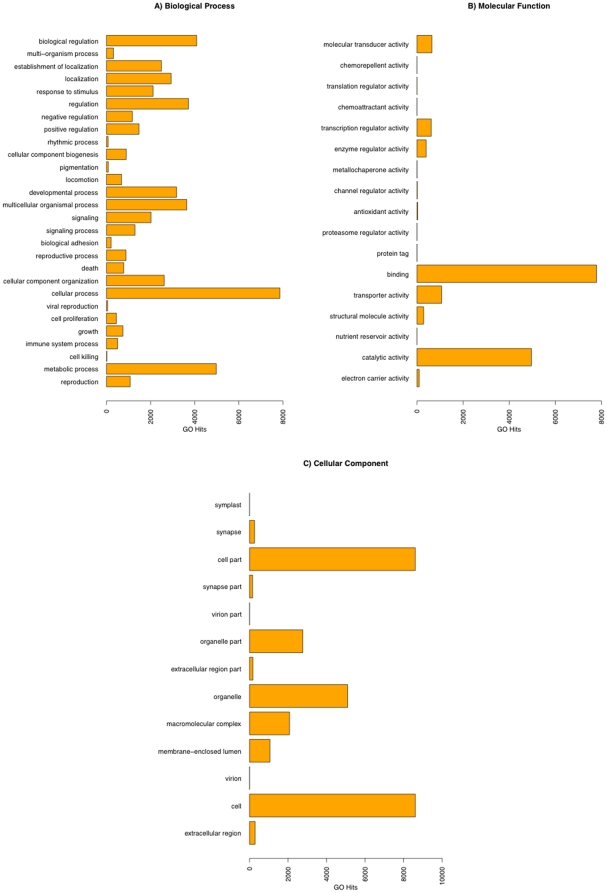
Gene ontology hits. The number of gene ontology (GO) hits for the second tier categories: (A) Biological process (B) Molecular function (C) Cellular component. More detailed GO term assignment is available as an additional file.

The transcripts were translated in all 6 frames into amino acid space and scanned for homology to Pfam-A protein families. This allowed 15,691 pfam family assignments to 11,597 transcripts. The Blast2go pipeline provided 73,491 GO term assignments to 10,803 transcripts. A further 13,724 assignments to 7,365 transcripts were obtained via pfam2go GO terms for Pfam-A families. These 2 datasets were condensed to 82,872 terms for 12,133 transcripts after accounting for redundancy between datasets. The hierarchical structure of GO terms allowed transcript classification to be summarised further to first tier terms of the three main domains of the GO database (Figure 8A, B, C).

**Figure 9:**
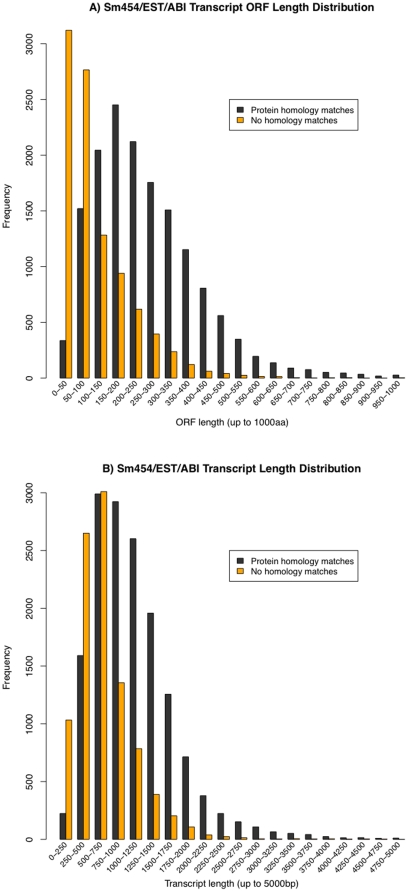
Distribution of ORF and transcript lengths with and without homology based annotation. Frequency plots of open reading frame length (A) and total transcript length separated by whether a transcript had homology (black) to another species by BLAST, PFAM and/or GO assignment or did not have any homology (orange) while shorter coding regions and transcript lengths are less likely to have homology this analysis suggests that many short transcripts and of the short ORFs (in the 0–50 aa range) are likely to be non-coding transcripts of the untranslated regions of non-coding transcripts.

Of the 25,053 total transcripts a total of 15,610 were classified by cross-species homology, a GO assignment, or pfam family homology, while 9443 remained unclassified (see Dataset S7 for GO assignments and PFAM annotations). An analysis comparing the distribution of transcript and open reading frame lengths of assigned and unassigned transcripts shows that the average classified transcript ORF length (266aa±182) is more than double that of the unclassified (111aa±98) (Figure 9A). Some of these unclassified short ORFs are likely to belong to short partial transcripts in our dataset (Fig 9B). The large number of transcripts with ORF less than 100 aa are likely to represent short fragments of protein coding transcripts, non-coding RNAs and possibly un-translated regions.

### An RNAseq approach to planarians defines the set of genes specifically affected by irradiation and therefore potentially expressed in stem cells and their recent progeny

**Figure 10:**
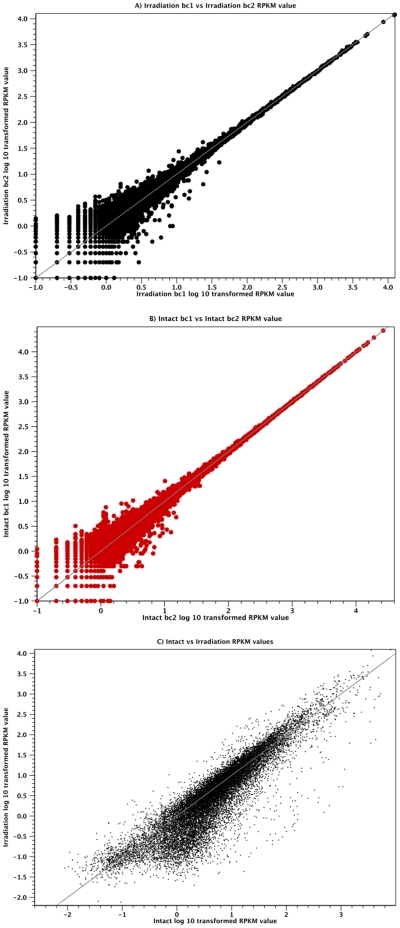
RNA seq expression values of Whole vs Irradiated expression. Plots show Log 10 transformed RPKM values for transcripts detected above an RPKM of >1 (Log 10 RPKM>0) in at least one sample. We compared the two replicates of RNAseq of irradiated worms (A) and intact (mock irradiated) to each other demonstrating a high level of congruency with differences in expression values only apparent at low expression levels. We then compared the replicates of irradiated worms against the replicates of intact worms (C) and are able to detect many differentially expressed transcripts, many of which decrease after irradiation. These represent a group of genes potentially expressed in planarian stem cell and their recent progeny.

We wished to establish the use of our dataset for the whole transcriptome analysis of expression levels in different experimental conditions, broadly called RNAseq [38]. We decided to use this approach to identify the set of transcripts that are likely to be enriched in proliferative stem cells and their recent progeny. Irradiation has been used extensively as an experimental tool to remove proliferating cells in planarians [46], [47]. Previous studies have utilized this to define sets of genes that are potentially expressed in pASCs and their progeny using microarrays and relatively limited gene sets [47], [48]. We performed further SOLiD 3+ sequencing on intact worms (mock irradiated) and worms 7 days after lethal irradiation (see materials and methods). This timepoint was chosen as it has previously been shown to remove both pASCs, their progeny that are no yet fully differentiated as well as possibly short lived differentiated cells [47]. We performed two replicates of each condition producing 13,392,444 and 31,688,899 mapped reads for intact worms and 10,334,419 and 11,372,969 reads for irradiated worms. We discarded transcripts for which we did not detect expression in all samples or where expression was close to zero in all samples to avoid the effects of low read sampling error on expression levels (RPKM<1). We analyzed expression levels between replicates and found a high congruency among replicates (Figure 10A and B).

We then pooled the results of each replicate experiment to compare in tact worms with 7 days irradiated worms (Figure 10C). We found a total of 2,304 transcripts that had differential expression (p-value less than 0.01, see Dataset S8 for FASTAs of this file, Dataset 9 containing information of P value, fold change and annotation of differentially regulated transcripts) and a decreased expression in irradiated samples of more than 2 fold, thus defining a definitive set of genes down regulated by irradiation treatment and potentially enriched in pASCs and there recent progeny (these are available in FASTA format as Dataset 8 and with fold down regulation, p-value and annotations as Dataset 9). The highest fold difference observed for any gene between irradiated and intact samples was a 459.64 fold decrease.Our findings are somewhat comparable to those of previous investigations using microarrays, though we have investigated many more genes [47], [48]. These transcripts are likely to fall into four categories: transcripts expressed in proliferative stem cells; transcripts expressed in recent stem cell progeny; transcripts that require the presence of proliferative stem cells for their expression but are expressed in differentiated cells, and finally, transcripts downregulated by irradiation irrespective of cell type expression profile. To validate our RNAseq results we looked at the response of genes that have been experimentally confirmed as having reduced expression after irradiation, as they are expressed in stem cells or their progeny, although some of them are also expressed in differentiated cell types (Table 3). We looked at 18 genes from different planarian species that have a role in pASC biology and/or their irradiation sensitive progeny, and have corroborating in situ hybridization data showing loss of expression after irradiation [47], [49], [50], [51], [52], [53], [54], [55], [56], [57], [58], [59], [60]. We found that in each case expression was significantly reduced (p<0.01, using the test of Baggerley et al [61] in irradiated samples and in 17/18 by at least two fold (Table 3). This suggests that our results are representative of the transcript set whose expression is affected by irradiation and are therefore candidates for potential roles in pASC biology.

**Table 3:** RPKM values of known genes in whole vs. irradiated worms.

Gene/EST and species	Accession	Reference	Sm454ESTABI Dataset ID	Mean Intact RPKM	MeanIrradiated RPKM	P-value	Fold-Change
Smedwi-1	DQ186985	[52]	9082	275.7	19.3	2.17E-121	−14.28
Smedwi-2	DQ186986	[52], [58]	20642	134.7	0.65	1.19E-77	− 207.23
Smed-bruli	DQ344977	[55]	2960	34.3	19.4	5.29E-03	−1.77
Smed-Histone-H2B	DN290330	[55]	17226	50.45	15.1	1.34E-8	−3.34
Smed-nanos-like	EF035555	[57]	24540	12.6	0.6	1.71E-07	−21
Dj-PCNA	EU856391	[59]	22122	46.85	2.35	9.25E-24	−19.94
Spol-Tudor	FJ655915	[50]	16133	49.25	20.65	1.36E-06	−2.38
Smed-HDAC	NP_004955	[47]	22368	190.25	94.9	3.65E-15	−2
Smed-NB.21.11.e	FG311845	[47]	17431	155.9	0.9	3.64E-89	−173.22
Smed-AGAT-1	AY967547	[47]	17877	95.75	0.8	3.2E-45	−118.75
Smed-p53	AY068713	[53]	19247	15.0	0.8	1.64E-8	−18.25
Dj-rrm2	AY067447	[48]	13945	70.2	8.55	1.34E-26	−8.21
smed-SMB	GU562964	[56]	19507	122	36.15	3.05E-23	−3.36
Smed-INX-11	Q851133	[54]	18619	50.2	13.4	1.34E-11	−3.75
Dj-Pumilo	AJ639658	[48], [49]	19575	72.25	28.55	6.22E-10	−2.53
Dj-EST, similar to H2Az)	BP186772	[48]	15734	47.55	1.4	5.39E-26	−33.96
DjEST, similar to TAF1-beta	BP187634	[48]	16295	584.95	207.15	1.42E-82	−2.82
Smed-CHD4	GU980571	[60]	4553	22.15	2.2	3.77E-10	−10.07

### Conclusions

We have successfully combined two different next generation sequencing platforms to produce a more complete view of the transcriptome of the planarian *S. mediterranea*. This dataset will help better define the genetic context of this model organism and allow the development of new reagents sets for 'omic approaches to understanding planarian biology. Of immediate significance is the ability to use our dataset combined with whole transcriptome sequencing to look at broadly and changes in expression. Here we have shown that this is possible by describing the set of transcripts that are down regulated by irradiation to remove proliferative stem cells and their recent progeny.

The transcriptome dataset present here defines many putative *S. mediterranea* proteins. Simple homology and ontological comparison of these proteins reveal an evolutionary pattern in agreement with our current view of the animal tree of life. *S. mediterranea* has added significance as a model system, beyond its regenerative capacity, due to its phylogetic position within the lophotrochozoan protostome group, an under represented group with respect to functional molecular studies.

Our dual platform approach to defining the transcriptome has a number of advantages over using just either a short read or long read platform alone. While a long read approach produces long reads that we can be confident represent real transcripts the ultra deep coverage afforded by short reads allows increased transcript discovery. Combining data from both platforms also likely alleviates platform biases specific to each workflow. As more and more animals genomes are sequenced using Next Generation sequencing platforms we are likely to have to work with genomes that although somewhat complete remain unassembled in many independent contigs. For these animals a dual platform transcriptome approach will be an affordable way to experimentally annotate these genomes with their cognate transcribed regions.

## Materials and Methods

### Animal culture and preparation of regenerative stages

A clonal line of the asexual strain of *Schmidtea mediterranea*, AAANOTBIOL01, was used for all experiments. Animals were reared at 20°C in tap water filtered through activated charcoal and buffered with 0.5 ml/L 1 M NaHCO_3_. Planarians were fed veal liver and starved for at least one week prior to experiments or amputation. All worms used were 7–8 mm in length.

Regenerating fragments for RNA preparation were collected and frozen at 6, 12, 24, 36, 48, and 72 hours, 96, 120 and 144 hours of anterior and posterior regeneration. 20 worms for each time point were cut in head, middle and tail fragments. Whole intact animals unfed for 7 days were also included as a separate sample.

### Preparation of RNA for sequencing

Planarian pieces were frozen at –80°C overnight in 300 µl Trizol (Invitrogen, Cat. No. 155966) and then RNA prepared using the manufacturers protocol. Samples were resuspended in nuclease-free water and treated with 2 U of Turbo DNase (Ambion, Cat. No. AM2238) in 1 x DNase buffer for 30 mins at 37°C. Samples were extracted with phenol, pH 4.3: chloroform (50∶50; Sigma, Cat. No. P4682-100 ML) and alcohol precipitated overnight at –80°C. Total RNA was pelleted by centrifugation at 12,000×g for 15 mins at 4°C, washed with 70% ethanol and air dried for 5 mins. RNA was resuspended in nuclease-free water.

### Preparation of 454 transcriptome libraries and sequencing

Concentration of total RNA for samples from each timepoint was measured with Qubit flourometer RNA assay kit (Invitrogen, Cat. No. Q32852). Equal amounts of total RNA from each stage was pooled together into a single sample. Ribominus eukaryotic kit (Invitrogen, Cat. No. A10837-08) was used according to manufacturer's instructions for depletion of ribosomal RNA. 1 µg of ribo-depleted RNA was used to make double stranded cDNA (dscDNA) with SMARTer PCR cDNA synthesis kit (Clontech, Cat. No. 634925) and normalisation of amplified dscDNA was carried out with the TRIMMER kit (Evrogen, Cat. No. NK001), according to the manufacturers instructions. 5 µg of dscDNA in 100 µl of TE was sonicated with a Covaris S2 in frequency sweeping mode at a water bath temperature of 5°C (fragmentation parameters: Duty cycle –5%, Intensity – 3, cycles/burst – 200 and Time – 120 secs). Following size fractionation with conventional gel electrophoresis, DNA fragments 400–900 bp were excised and purified with QIAquick gel extraction columns (Qiagen, Cat. No. 28760). Finally, gel purified dscDNA was used to create a fragment library as described in the Roche GS FLX titanium general library preparation guide. Sequence was performed on Roche 454 sequencing machine according to the manufacturers instructions. All data is available at the NCBI short read archive under study number SRP002478.

### Preparation and sequencing of SOLiD 3 whole transcriptome libraries

Pooled total RNA from regenerative stages and in tact worms was enriched for mRNA enriched using the Poly A Purist Kit (Ambion, Cat. No. AM1919) followed by further depletion of ribosomal RNA using the Ribominus Eukaryotic kit (Invitrogen, Cat. No. A10837-08). Solid whole transcriptome libraries were made as outlined in the Solid Whole transcriptome kit protocol (Applied Biosystems, Cat. No. 4425680). The Quant-it HS dsDNA assay kit (Invitrogen, Cat. No. Q32851) was used to measure the concentration of libraries. Sequencing was performed on a SOLiD 3 ABi sequencer according to the manufacturers instructions to generate 50 bp reads in colour space. All data is available at the NCBI short read archive under study number SRP002478.

### Assembly of 454 reads

Roche's de novo GS Assembler (Newbler 2.3) was used to assemble reads from the 454 platform. Newbler produces the assembly in FASTA format along with several metric files. The Newbler algorithm allows discovery of potential isoforms which are contained in the output as ‘isotigs’. The publicly available ESTs for *S. meidterranea* were also added to the assembly and the resulting sequences are available in the Sm454EST.fa FASTA file.

### Mapping and assembly of SOLiD 3 reads

The ABi transcriptome dataset was generated with an iterative mapping approach. Reads from the ABi SOLiD 3 platform was mapped using the Whole-Transcriptome Pipeline (WTP) within the BioScope 1.0 software package. The WTP of this mapping pipeline does not produce de novo split reads that span possible introns. It requires gene structure definitions in the form of a GTF formatted file to find possible split reads. We used MAKER gene annotations from SmedGD as the input gene definitions for WTP. The mapping produced a SAM formatted file containing mapping coordinates, mapping quality, and information on split reads. The Cufflinks program [41] was used define transcribed regions using the SAM mapping file as the input. We used the following parameters optimized to give correct coverage of a defined set of planarian genes that have been experimentally cloned and are present in the NCBI database. On this basis Cufflinks was run with the following parameters: Q 100 -I 100000 -F 0 -f 0.3 -j 0.1. Cufflinks based assembly was performed using only high quality uniquely mapped reads. The assembly produces a GTF gene annotation file of transcribed regions with all potentially transcribed regions, we required that these be represented by at least 2 uniquely mapped high quality reads.

Cufflinks uses split-reads to lace together exons into a genes and to define isoforms. In order to define as many possible split-reads as possible, a new gene structure definition file was created using the assembled GTF file. This file contains all assembled genes on a genomic contig laced together as one ‘super-gene’. One super-gene was created for each strand of the genomic contig. This new pseudogene definition file was then used for remapping using WTP with only high quality reads that had previously only failed to map or only mapped along part of their length.

From the new SAM mapping file, all new split-reads were extracted and added to the initial SAM file. A re-assembly was done using Cufflinks on this new SAM file with more split-reads resulting in a dataset with significantly more multiple-exon genes. Putative transcripts were then extracted as cDNA sequences with introns removed. This data is available as the SmABi.fa FASTA file.

### Comparison of datasets with Smed GD MAKER annotations

Comparison of Sm454EST, SmABI, Sm454ESTABI, and SmMAKER was done using BLASTN with the e-value threshold set at 1E-10. All three dataset fasta files were concatenated into one file and an all versus all blast was done. The blast results were then parsed, categorized, and filtered for only blast matches that had 95% or better identity.

### Merging of datasets from 454 and SOLiD

The 454 dataset was used as a scaffold for the ABi dataset. The 454 assembly from Newbler contains possible isoforms. The isoforms with only terminal differences at the 3′ and 5′ ends were collapsed by running the dataset through CAP3 assembler [42]. The ABi dataset is was then blasted (BLASTN) against this collapsed 454 dataset. A list of blast matches and a list of non-matches are generated by filtering the blast results (BLASTN with the e-value threshold set at 1E-10, and filtered for only blast matches that had 95% or better identity across 50 nucleotides or more). Out of the list with non-matches, any ABi transcripts that had multiple exon structures were maintained. Finally, ABi transcripts with BLAST match(es) and ABi transcripts that made multiple exons with no BLAST matches were assembled with the 454 dataset with CAP3 assembler. This results in a list of assembled contigs and a list of singlets. From the list of singlets, any transcript that was only represented single exon ABi SOLiD data was removed. The assembled contigs and filtered singlets list is then concatenated together as the merged dataset resulting in 25,0053 putative transcripts. These are available in the Sm454ESTABI.fa.txt FASTA file (Dataset S4).

### Open Reading Frame analysis

We used CLC Genomics Workbench (www.clcbio.com) for open reading frame analysis. ORFs were allowed to start and end outside the given sequence. Putiative peptides are available in the file Smed454ESTABI.orf.fa.txt FASTA file (Dataset S5)

### Gene ontology and PFAM analysis

The complete six-frame amino acid translations of each transcript were tested for homology to Pfam-A protein families using the hmmscan algorithm applied to the Hidden Markov Model dataset (Pfam-A.hmm v.24) [43]. An e-value threshold of 1E-5 was applied to determine family homology of each transcript. Gene Ontology (GO) terms where assigned to Pfam matched transcripts via the pfam2go lookup table (http://www.geneontology.org/external2go/pfam2go). This procedure was implemented using a Perl script.

GO terms were also assigned to transcripts using the Blast2go pipeline version 2.3.5 [45]. Transcript sequences were compared initially to a BLAST database comprising the NCBI invertebrate non-redundant (nr) protein dataset supplemented with the proteome sequences used in the cross-species homology analysis. Sequence redundancy was removed according to unique GenBank Identification (GI) code. A lower e-value threshold of 1e-5 was used to determine significant homology matches and results were recorded in XML format. GO terms where then assigned to each transcript through automated queries to the Blast2go MySQL term tables. The resulting Blast2go annotations where then added to those defined by Pfam2go with duplicate GO term assignments removed. The classifications for individual transcripts are presented as features in Dataset S6. Determining the total number of transcripts classified according to first tier terms of the three main domains of the GO database was achieved using a Perl script querying the Blast2go MySQL term tables.

### Analysis of homology with other animals

The number of protein sequences defined for each species used in the cross-species homology analysis were as follows: *Strongylocentrotus purpuratus*: 42420, *Homo sapiens*: 37391, *Mus musculus*: 34962, *Arabidopsis thaliana*: 33049, *Branchiostoma floridae*: 28623, *Caenorhabditis elegans*: 23894, *Drosophila melanogaster*: 17702, *Hydra magnipapillata*: 17398, *Ciona intestinalis*: 13842, *Schistosoma mansoni*: 12856, *Anopheles gambiae*: 12659, *Brugia malayi*: 11470, *Acyrthosiphon pisum*: 10466, *Tribolium castaneum*: 9833, *Nasonia vitripennis*: 9254, *Apis mellifera*: 9244, *Schizosaccharomyces pombe*: 5000, *Saccharomyces cerevisiae*: 4027. Proteome sequences were sourced from NCBI except those of *Schistosoma mansoni*; sourced from GeneDB [62]. Transcript sequences where compared to the blast database of the proteins using BLASTX. A minimum e-value threshold of 1e-5 was applied and results were recorded in XML format. Default values for all other parameters where used. Blast results where processed using scripts implementing BioPerl modules. The protein sequence conferring the closest match to each transcript was determined by E-value, then by bit score. These matches are also presented as features in Dataset S6.

### RNAseq analysis of irradiated versus whole worms

Two batches of 10 whole worms were starved for one week prior to gamma irradiation, exactly as previously described. Two batches of matched controls were mock irradiated. RNA from all four batches was prepared as described above and used to make four separate SOLiD 3 transcriptome libraries (see above). These were then sequenced as four separate barcodes on one flow cell to generate 50 bp color space reads. Reads were mapped to the Sm454ESTABI transcriptome with Bioscope version 1.0 WTO pipeline. The number of unique tags per transcript for each sample were counted and converted into Reads per KB per million (RPKM) values. These are values normalized by number of reads and gene length.

We used the statistical package for gene expression analysis within the CLC BIO Genomics Workbench (www.clcbio.com) to analyze gene expression levels between replicates and between intact and irradiated worms (p>0.01, using the test of Baggerey et al [61]). We did not consider genes that had very low expression levels across all 4 samples (RPKM<1, Log10 of RPKM<0).

We specifically identified transcripts representing genes that have been experimentally confirmed in published literature as being expressed in neoblast stem cells or their progeny and compared their RPKMs between whole and irradiated worms to evaluate our dataset (Table 3).

## Supporting Information

Dataset S1 TheSm454EST dataset in FASTA format is an assembly fof 454 reads generated in this work and publicly available ESTs.
(TXT)

Dataset S2 The SmMAKER dataset in FASTA format.
(TXT)

Dataset S3 The SmABI datset in FASTA format.
(TXT)

Dataset S4 Sm454ESTABI dataset iN FASTA format.
(TXT)

Dataset S5 The longest predicted open reading frames of the Sm454ESTABI dataset in FASTA format with positional coordinates and strand information along the coordinates
(TXT)

Dataset S6 The data file detailing the cross species BLAST results top hits.
(TXT)

Dataset S7 The data file detailing all of the GO and PFAM annotations of the Sm454ESTABI dataset.
(TXT)

Dataset S8 The data file containing the FASTA files of transcripts differentially regulated after irradiation.
(TXT)

Dataset S9 Data file containing P-values, fold change, BLAST and GO information for transcripts differentially regulated after irradiation.
(TXT)
